# Integrating circulating-free DNA (cfDNA) analysis into clinical practice: opportunities and challenges

**DOI:** 10.1038/s41416-022-01776-9

**Published:** 2022-03-26

**Authors:** Miguel García-Pardo, Maisam Makarem, Janice J. N. Li, Deirdre Kelly, Natasha B. Leighl

**Affiliations:** Princess Margaret Cancer Centre, University Health Network, Toronto, ON Canada

**Keywords:** Cancer, Biomarkers

## Abstract

In the current era of precision medicine, the identification of genomic alterations has revolutionised the management of patients with solid tumours. Recent advances in the detection and characterisation of circulating tumour DNA (ctDNA) have enabled the integration of liquid biopsy into clinical practice for molecular profiling. ctDNA has also emerged as a promising biomarker for prognostication, monitoring disease response, detection of minimal residual disease and early diagnosis. In this Review, we discuss current and future clinical applications of ctDNA primarily in non-small cell lung cancer in addition to other solid tumours.

## Liquid biopsy in oncology

Peripheral blood analysis to detect circulating-free DNA (cfDNA) and circulating-tumour DNA (ctDNA) has been extensively studied, and technologies that detect genomic aberrations and quantify DNA in circulation are rapidly evolving. Research efforts in this field are becoming of interest to clinicians world-wide and several comprehensive reviews have reviewed the advances in methodology, analytical validity, and future directions of liquid biopsy technologies [1–4]. Although tissue biopsy is still necessary to obtain histologic diagnosis, PD-L1 evaluation, and other relevant biomarkers, the use of ctDNA is becoming more prevalent in clinical settings, as a plasma-first, complementary or sequential approach to tissue testing. The integration of liquid biopsy in clinical practice has been increasingly studied in non-small cell lung cancer (NSCLC) [5]. In this review, we discuss the current and future applications of ctDNA in clinical practice, with a focus on clinical utility in NSCLC and a select number of additional solid tumours.

ctDNA analysis offers a non-invasive approach for initial diagnosis and longitudinal treatment response, by capturing tumour heterogeneity and resistance patterns. Although powerful single cell sequencing technologies are at the forefront of understanding tumour biology [6], tissue samples from serial biopsies of patient tumours seldom capture the genomic complexity of the entire tumour and metastatic sites. Therefore, liquid biopsy offers an advantage over tissue biopsy by capturing inter- and intra-tumour heterogeneity of tumours, especially in the metastatic setting [7, 8]. Currently, cfDNA, of which ctDNA is a small fraction, is the most common analyte studied from liquid biopsies and is commonly integrated into clinical practice. ctDNA can be found in a variety of fluids, including pleural or peritoneal fluid, or cerebrospinal fluid (CSF) [9–11], but plasma remains the preferred source [12].

**Fig. 1:**
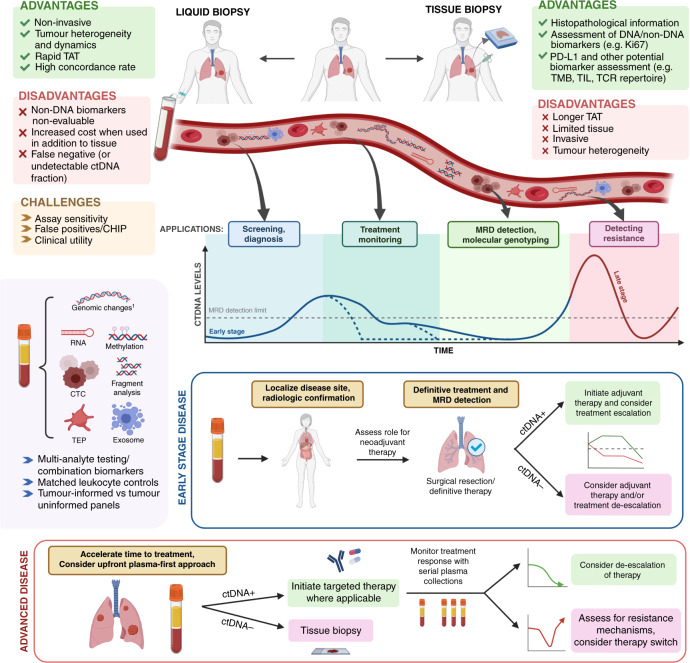
Opportunities and challenges of ctDNA in cancer. The advantages and disadvantages of utilising ctDNA technology compared with tissue biopsy are highlighted in green and red boxes, respectively. The graph demonstrates varying ctDNA kinetics. Dotted blue lines demonstrate potential cases of early ctDNA clearance. ctDNA+: ctDNA positive result, ctDNA−: ctDNA negative result. ^1^Genomic changes refer to point mutations, gene re-arrangements, insertions/deletions, or copy number changes. Figure created with BioRender.com. TAT turn around time, TMB tumour mutational burden, TIL tumour-infiltrating lymphocytes, TCR T-cell receptor, CHIP Clonal hematopoiesis of indeterminate potential, CTC circulating tumour cell, TEP tumour-educated platelet, MRD minimal residual disease.

In addition to ctDNA, circulating tumour cells (CTCs), RNA (circulating free RNA, non-coding RNAs), tumour-educated platelets (TEPs) and extracellular vesicles/exosomes can also be isolated and have recently demonstrated relevant mechanisms through which they can modify or be modified by tumour cells leading to predictive and prognostic biomarkers (Fig. 1) [13, 14]. There has been recent data showing that CTCs may also be useful predictive markers of recurrence and survival in patients with solid tumours [15, 16]. In patients with metastatic breast cancer, several studies have shown that the number of CTCs at baseline is an independent predictor of progression-free survival (PFS) and overall survival (OS) [16, 17]. Notably, several phase II and phase III clinical trials such as TREAT-CTC, STIC-CTC and ongoing trials have shed light onto the clinical utility of this cell population [18, 19]. In lung cancer, the combination of cfDNA analysis and CTCs for *EGFR* mutation evaluation using VTX-1 liquid biopsy system (Vortex Biosciences) has enabled the analysis of *EGFR* mutations in both CTCs and cfDNA from a single blood collection [20, 21]. However, characterisation of CTCs that contribute to metastasis remains elusive, owing to their phenotypic heterogeneity. Adoption of CTC use or other non-ctDNA based plasma biomarkers in routine clinical practice will require robust demonstration of analytic validity, clinical validity, and, most importantly, clinical utility [22, 23]. This review will focus on the clinical applications of ctDNA.

## Current applications

### Molecular genotyping of advanced disease

Targeting the activation of specific molecular pathways to inhibit tumour growth has led to personalised treatments with significant clinical and survival benefit [24–26]. In the current era of targeted therapy, the detection and characterisation of genomic alterations that drive tumour growth has prompted several guideline-based recommendations for many actionable targets for drug therapy that improve patient outcomes (Table 1).

**Table 1:** Current recommendations for ctDNA analysis in solid tumours.

	IASLC	ASCO/CAP	ESMO NSCLC	NCCN – NSCLC	NCCN- Breast cancer	NCCN—Oesophageal, EJG, and gastric	NCCN - Melanoma
Plasma over serum	✓	✓					
Prioritise histologic diagnosis	✓	✓		✓			
If tissue insufficient, medically unfit, or expected delay, consider plasma testing	✓			✓		✓	✓
Plasma genotyping at progression to detect targetable alteration	✓	✓	✓	✓	✓	✓	
If plasma negative, tissue biopsy recommended	✓	✓	✓	✓	✓	✓	
A positive, actionable ctDNA-detected alteration is sufficient to initiate treatment	✓	✓	✓	✓	✓	✓	
NGS is preferred for detecting fusions	✓						
Reports to include platform used and molecular findings	✓	✓					
Establish analytical validity of each assay	✓	✓		✓			
Account for CHIP-related alterations	✓	✓		✓			

Check marks indicate recommendations based on NCCN guidelines (tumour-specific).

*NSCLC* non-small cell lung cancer, *CHIP* Clonal hematopoiesis of indeterminate potential, *EGJ* Esophagogastric, *IASLC* International Association for the Study of Lung Cancer, *ESMO* European Society for Medical Oncology, *ASCO/CAP* American Society of Clinical Oncology, College of American Pathologists.

The European Society of Medical Oncology (ESMO) recommends routine use of broad panel next-generation sequencing (NGS) on tumour samples in advanced non-squamous NSCLC, prostate cancer, ovarian cancer and cholangiocarcinoma [27]. Although the gold standard for molecular testing is tumour tissue genotyping, ctDNA analysis has demonstrated clinical utility as an alternative or complementary tool in NSCLC, especially in clinical scenarios where tissue or time is limited [28–30]. Currently, the National Comprehensive Cancer Network (NCCN) recommends plasma-based molecular testing as part of broad molecular profiling in all patients with non-squamous NSCLC at the time of diagnosis, if there is insufficient tissue to test all relevant targets, (*EGFR*, *ALK*, *ROS1*, *BRAF*, *MET*, *NTRK* and *RET)* [31]. Plasma-based testing is also preferred for patients who are medically unfit for repeat tumour biopsy. Additionally, the International Association of the Study of Lung Cancer (IASLC) consensus statement in 2018 concluded that ctDNA approaches have significant potential to improve patient care, and immediate implementation in the clinic is justified in a number of therapeutic settings in NSCLC including in the diagnosis of EGFR tyrosine kinase inhibitor (TKI) resistance (*EGFR* T790M) [28].

#### NSCLC

The most comprehensive evidence for blood-based ctDNA analysis for molecular genotyping has stemmed from studies in advanced NSCLC. The NILE (Noninvasive vs Invasive Lung Evaluation) clinical trial (NCT03615443) prospectively analysed the clinical utility of plasma-based NGS (Guardant360™) for first-line genotyping in patients with metastatic non-small cell lung cancer (NSCLC) as compared with tissue genotyping. In 282 patients analysed, the Guardant360™ NGS assay detected clinically relevant NSCLC-associated biomarkers at a similar rate to standard-of-care (SOC) testing and was deemed non-inferior. The combination of tissue-based and plasma-based genotyping and cfDNA resulted in a higher frequency of driver mutations identified than either method alone. In addition, the median turn around time for plasma-based NGS was significantly lower than for SOC tumour genotyping (9 days vs. 15 days) [29].

In a cohort of 229 patients, Aggarwal et al. has also demonstrated that adding plasma NGS to the routine management of patients with metastatic NSCLC increases targetable mutation detection and improves delivery of molecularly-guided therapies [30]. More recently, another prospective study using InVisionFirst™ also demonstrated excellent concordance with tissue profiling in 264 patients with untreated advanced NSCLC [32]. The Blood First Assay Screening Trial (BFAST) is an ongoing multi-cohort study (NCT03178552) evaluating plasma-based NGS (FoundationOne Liquid) for detection of actionable genomic alterations in patients with advanced NSCLC, and assessing the efficacy of targeted and immunotherapy-based therapies on plasma results alone. Preliminary data from patients with *ALK*-fusion positive disease showed a high overall response rate (ORR) and clinical benefit in patients with *ALK* fusions detected in plasma and treated with alectinib [33, 34]. A growing number of registration studies of novel agents such as capmatinib, tepotinib and sotorasib have also used liquid biopsy as an initial diagnostic method for target identification, leading to broader regulatory acceptance of ctDNA as a diagnostic tool in patients with advanced NSCLC [35–37]. These studies have validated the clinical utility of blood-based NGS to guide precision medicine in patients with advanced NSCLC at diagnosis.

#### Other advanced solid tumours

In HER2-negative, hormone receptor-positive advanced breast cancer, SOLAR-1 demonstrated that *PIK3CA* mutations detected in tissue or ctDNA can guide treatment with alpelisib in combination with fulvestrant, after progression on prior endocrine therapy [38, 39]. This trial demonstrated improved PFS in patients with *PIK3CA* mutations detected in plasma ctDNA [40]. NCCN Guidelines for invasive breast cancer (version 2.2022) recommend assessment for *PIK3CA* mutations in tumour tissue or ctDNA in this setting [41]. However, in line with current state-of-the-art molecular genotyping in NSCLC, a negative *PIK3CA* plasma test should be followed by profiling in tumour tissue due to assay sensitivities. Additionally, the TAPUR study demonstrated that single-agent pembrolizumab has meaningful clinical activity in heavily pretreated patients with metastatic breast cancer with high tumour mutational burden (TMB) detected in tissue or blood [42].

In metastatic colorectal cancer (mCRC), *KRAS*, *NRAS* and *BRAF* assessment is mandatory for treatment selection and prognostication, as *RAS* mutations confer resistance to anti-EGFR antibodies and *BRAF* V600 mutations associate with poor prognosis. In this setting, ctDNA testing using single gene assays (PCR-based) has been approved as an alternative to tissue-based genotyping [43, 44]. Currently, several trials are exploring the clinical utility of ctDNA analysis in mCRC in different settings. The CHRONOS trial (NCT03227926) explored the role of ctDNA analysis to select patients for rechallenge with panitumumab in the third line setting in patients with *RAS* wildtype mCRC. Of 52 patients tested, 69% had no ctDNA evidence of *RAS/RAF/EGFR* mutations. Re-challenge panitumumab was associated with an ORR of 30%, demonstrating the potential of ctDNA to identify patients for successful retreatment [45].

In patients with metastatic castration-resistant prostate cancer, ctDNA analysis to detect *BRCA*1 and 2 mutations can guide treatment with PARP inhibitors [46]. There is high concordance of mutation detection in plasma and tumour tissue [47, 48]. As a result, FoundationOne Liquid™ is approved by the US FDA for these indications [49]. In melanoma, the use of single gene (BEAMing) assays to detect *BRAF* V600E mutations in plasma has been challenged by a low sensitivity rate of 75% [50]. However, research is ongoing in the area of prognostication and response monitoring. Other single gene detection methods have been developed in order to analyse *BRAF* status in blood samples, such as Allele-specific amplification Refractory Mutation System PCR (ARMS), Allele-Specific PCR (AS-PCR), PNA-PCR clamping technique, and digital droplet PCR (ddPCR) [51].

Finally, clinical utility of ctDNA detection is also being investigated in gastroesophageal adenocarcinoma (GEA). NCCN guidelines recommend plasma-based NGS if a patient is unable to undergo a tissue biopsy [52]. Several studies have explored the role of plasma ctDNA NGS in guiding clinical decision-making in GEA [53, 54]. Targetable mutations were identified at higher frequency using ctDNA compared with previous tissue NGS testing, including detection of microsatellite instability (MSI) and *HER2* amplification.

### Detecting acquired resistance

In the advanced clinical setting, the identification of specific resistance mechanisms can inform subsequent therapies, paving the way for implementation of ctDNA-based methods to guide therapy. Subsequent to the development of resistance to first- or second-generation EGFR TKIs in *EGFR* mutant advanced NSCLC, patients benefit from osimertinib in the second-line setting if a *EGFR* T790M resistant mutation is identified [55]. Several studies have shown the clinical utility of T790M detection in ctDNA using various platforms leading to implementation of liquid biopsy in this clinical setting. For patients with a plasma T790M-negative result, reflex tissue biopsy and tissue genotyping is recommended [56–58]. Osimertinib, a third-generation EGFR inhibitor, has now moved to the first-line setting as treatment for patients with advanced *EGFR* mutant NSCLC [24]. Although resistance via T790M has been described with this agent, ctDNA testing is now being explored as a tool to identify novel resistance mechanisms to guide future treatment [59]. Mechanisms of resistance have included *MET* amplification and *EGFR* C797S mutations, *HER2* amplifications, among others, all of which can be detected in ctDNA [60, 61]. Plasma-based NGS has also demonstrated clinical utility in detecting targetable alterations and characterising resistance mechanisms after progression with ALK inhibitors in *ALK*-rearranged NSCLC patients [62, 63]. If a single or limited number of resistance mechanisms is expected, PCR-based single gene assays may be advantageous. However, in most settings, comprehensive NGS panels are more likely to identify potential resistance alterations and are becoming a routine adjunct to tissue biopsy. If an actionable target is identified in ctDNA, this is considered sufficient to direct therapy (Table 1).

We anticipate that ctDNA analysis will be integrated broadly as a complementary genomic diagnostic method not only at initial diagnosis, but to characterise resistance mechanisms to targeted therapies and inform sequential therapy.

Nevertheless, clinicians should also consider histological transformation from NSCLC to small-cell lung cancer as a potential mechanism of resistance to targeted therapies [64], which would not be captured by ctDNA analysis. In certain clinical settings (i.e. extensive tumour growth, loss of *Rb1* and *p53* detected in ctDNA), tissue biopsy should be pursued [65].

## Opportunities: emerging landscape and future clinical applications

In addition to molecular genotyping in advanced solid malignancies and aiding in treatment selection at resistance, ctDNA is emerging as a promising biomarker in other clinical scenarios such as cancer screening and monitoring treatment response. There is mounting evidence for the use of ctDNA as a marker of prognosis, treatment response, as well as detection of minimal residual disease (MRD) and relapse risk after curative therapy. However, prospective trials to demonstrate the clinical utility of ctDNA in these settings are still needed. Clinical utility is the measure of a test’s ability to result in better patient outcomes through improved treatment selection.

### Accelerate time to treatment in advanced disease

Given the major impact of wait times for molecular testing results on patient outcomes, liquid biopsy can be used to accelerate the molecular diagnosis and time to treatment of patients with advanced cancer (Fig. 1). It is currently being investigated in prospective trials in patients with radiographic evidence of advanced lung cancer, prior to diagnostic tissue biopsy and profiling, with encouraging results [66, 67]. In a cohort of 55 patients with suspected advanced lung cancer, plasma-based molecular profiling performed at the time of diagnostic biopsy was highly concordant with tissue sequencing and was associated with shorter time to treatment initiation [67]. In a similar study in patients with suspected advanced lung cancer, 22% of patients commenced targeted therapy as a result of plasma-based molecular profiling without tissue molecular results; blood-first ctDNA NGS increased the rapidity of detection of actionable alterations with high tissue concordance, leading to timely treatment decisions [66]. These studies aim to shorten the time to treatment for patients with and without targetable alterations (NCT04863924). However, “plasma first” does not mean plasma only; tumour biopsy is required for lung cancer diagnosis, pathologic subtyping, and PD-L1 assessment in advanced disease [68].

### Blood-based predictive biomarkers for cancer immunotherapy

Optimal selection of patients for immunotherapy remains a challenge. The use of PD-L1 as a biomarker has limitations, given its limited predictive value in certain tumour types. Several studies are exploring additional biomarkers in tissue and blood to identify those who may benefit most from treatment. Given the clinical benefit of pembrolizumab seen across several tumour types in patients who are MSI-H or have high TMB (≥10 mut/Mb) as seen in the KEYNOTE 158 trial [69–71], the US FDA granted approval for pembrolizumab in this setting [72–74]. This has led to studies exploring whether ctDNA detection of these biomarkers in blood (bTMB) can predict response to check-point inhibitors. Non-invasive approaches for detection of MSI and TMB in plasma appear feasible and can overcome sampling bias seen with tissue sampling [75, 76]. ctDNA analysis can be used for TMB measurement as a surrogate of tissue-based TMB. In the MYSTIC phase III trial, ctDNA for quantifying bTMB from plasma appeared to predict clinical benefit with durvalumab and tremelimumab compared with conventional chemotherapy in patients with NSCLC [77]. Analysis of patient samples from the OAK study also demonstrated that bTMB can predict PFS with atezolizumab [78], and appeared to be an independent biomarker from PD-L1 status [79]. A large real world analysis of almost 30,000 patients demonstrated that those with various GI malignancies who underwent ctDNA analysis using Guardant 360 to identify MSI-H status derived clinical benefit from immune checkpoint inhibition [80]. A few limitations do exist given that tumours need to shed enough ctDNA for the assays to be valid, as such, prospective validation studies are required before broader implementation.

The tumour microenvironment can also provide important clues as to how an individual tumour and patient may respond to immunotherapy. Recently, this has included the T-cell receptor (TCR) repertoire of tumour-infiltrating lymphocytes [81–83]. TCR repertoire diversity in peripheral blood prior to treatment initiation could also represent a predictive biomarker to guide the use of immunotherapy [84, 85].

### ctDNA dynamics: prognostic impact and treatment response monitoring in advanced disease

Monitoring treatment response to therapy is another emerging application of ctDNA technologies, which may be relevant to all forms of therapy including chemotherapy, immunotherapy, targeted therapy, radiation therapy and surgery.

#### NSCLC

In NSCLC and other solid tumours, ctDNA detection is being explored as a prognostic marker and surrogate for monitoring treatment response. Detection of ctDNA in plasma appears to correlate with tumour burden [86]; pre-treatment levels of ctDNA may predict long-term survival in locally advanced NSCLC [87, 88]. Early ctDNA changes can be detected at first follow-up, prior to radiographic response [89]. Anagnostou et al. demonstrated that ctDNA response precedes radiologic response by an average of 9 weeks, making it a potential tool to monitor and guide patient therapy [90]. Plasma ctDNA clearance or decreasing levels have also been associated with improved response rates, PFS and OS [91].

In the targeted therapy setting, Gray et al. demonstrated that treatment-naïve patients with advanced *EGFR* mutant NSCLC without evidence of plasma ctDNA at diagnosis had improved PFS when treated with osimertinib, likely related to lower tumour burden [92]. Early clearance of plasma *EGFR* mutant clones was also predictive of response to osimertinib in the AURA-3 trial [93]. In a large real-world prospective cohort of 949 patients with advanced NSCLC carrying driver mutations, higher plasma ctDNA levels were associated with poor prognosis, and similarly clearance of ctDNA with targeted therapy was associated with better PFS and OS [79].

In patients with NSCLC receiving first line immunotherapy or combination immunotherapy and chemotherapy (if PD-L1 < 50%), a retrospective study evaluated the role of early ctDNA changes in predicting response to therapy. Those with a decrease in allelic frequency of patient-specific mutations had evidence of a radiographic response and longer median PFS and OS, especially if that decrease was detected after the first on-treatment blood draw, and the converse was evident, where increases in allelic frequencies appeared to correlate with progressive disease [91]. Nabet et al. explored pre-treatment ctDNA and peripheral blood T cell levels, and both were independently associated with immunotherapy response in NSCLC. Additionally, early ctDNA dynamics after a single infusion identified patients who achieved durable clinical benefit from immunotherapy [94].

Hellman et al. explored the association of plasma ctDNA and risk of disease progression in patients with advanced NSCLC with long-term administration of PD-(L)1 inhibitors. Of 27 patients with undetectable plasma ctDNA, 25 (93%) remained progression-free, whereas 4 patients with detectable plasma ctDNA developed disease progression [95].

ctDNA clearance appears to correlate with clinical response and benefit in patients treated with immune checkpoint inhibition. This has been demonstrated in a prospective Phase II trial to assess ctDNA in five cohorts of patients with advanced solid tumours treated with pembrolizumab (NCT02644369). Using a bespoke amplicon-based ctDNA detection platform, Bratman et al. demonstrated that serial analysis of ctDNA kinetics can predict response to immune check points inhibitors, with clearance of ctDNA preceding radiographic responses, and predicting favourable outcomes [96].

The identification of transformative immunotherapy biomarkers that can predict which patients may benefit most from immunotherapy remains a challenge. Determining whether ctDNA levels can be used to direct subsequent therapy or discontinuation of therapy will be an important next step to validate the clinical utility of this observation.

#### Response monitoring in other solid tumours

The potential utility of ctDNA dynamics for prognostication and tumour monitoring has been explored in other solid tumours as well. Salvianti et al. demonstrated the ability of ctDNA testing to detect disease progression earlier than standard monitoring in a cohort of patients including 20 with *KRAS* mutant mCRC [97]. A larger study of 43 patients with mCRC used a plasma-based NGS panel to monitor ctDNA changes in patients receiving first-line chemotherapy. The dynamic change of plasma mutation status was consistent with tumour burden and was closely correlated with disease progression. Interestingly, the concordance of mutation status between ctDNA and tumour samples was low at 54.6% [98].

In patients with metastatic breast cancer, early ctDNA dynamics can predict response to CDK4/6 inhibitors plus endocrine therapy [99, 100]. ctDNA at baseline has also been shown to be a useful prognostic marker in patients with advanced melanoma [101, 102]. Those with metastatic BRAF V600E mutant melanoma treated with targeted therapy in the COMBI-d study (NCT01584648) had prolonged PFS and OS if ctDNA was cleared at week 4 after treatment initiation [103].

### Clinical applications in early-stage disease

#### Prognostic role of ctDNA in early-stage NSCLC

Pre-operative ctDNA detection can identify patients at higher risk of disease recurrence [104]. In patients with stage I-III NSCLC who went on to surgical resection, pre-operative ctDNA levels were associated with positive clinical outcomes. Patients with detectable pre-operative ctDNA had significantly shorter recurrence-free survival compared to patients with undetectable ctDNA levels prior to surgery. The presence of plasma ctDNA prior to surgery likely reflects the presence of micrometastatic disease. Such patients may benefit from neoadjuvant or adjuvant therapy; however, randomised, prospective trials are needed to demonstrate clinical utility.

#### ctDNA dynamics and preoperative NSCLC therapy

Recent studies have explored the role of novel therapies in the preoperative setting, including targeted and immunotherapy. In advanced NSCLC, adding immune checkpoint inhibitors to chemotherapy improves survival [105]. When moved to the preoperative setting, combination immunotherapy with chemotherapy significantly increases the rate of pathologic complete response and ctDNA clearance, which appear to correlate [106]. However, how this will translate to event-free survival is unknown and must be demonstrated before ctDNA clearance will be accepted as a surrogate endpoint in early-stage disease.

#### Minimal residual disease (MRD) after curative intent treatment—NSCLC

Adjuvant therapy has demonstrated a modest survival benefit in several tumour types including NSCLC [107, 108]. ADAURA, a randomised phase 3 clinical trial, demonstrated a significant disease-free survival benefit in patients with resected stage IB to IIIA *EGFR* mutation–positive NSCLC treated with adjuvant osimertinib compared those who received placebo [109]. However, current methods used to identify those at risk for recurrent disease after curative therapy remain imperfect. Thus, the detection of MRD after curative therapy may be an opportunity to better select patients that may benefit from further adjuvant therapy or those who can omit treatment without impacting survival outcomes.

In a series of 40 patients with resected NSCLC, Chaudhuri et al. reported that 94% of patients with post-operative recurrence had ctDNA identified in their first post-operative blood sample [110]. This preceded radiographic progression by 5.2 months in most (72%) patients. Recent updates from the TRACERx study show that plasma ctDNA was detected at or before the time of clinical relapse in 38 of 42 patients [111]. The median time to ctDNA detection was 164 days while the median time to clinical relapse was 362 days. Similarly, ctDNA levels were measured pre- and post-resection for patients with stage IA-IIIA NSCLC as part of the LUCID study [112, 113]. Approximately 72% of patients had ctDNA detectable at baseline or in follow-up at levels as low as 6 ppm, with a lead time of 6 to 12 months before clinical relapse. The detection of ctDNA post-operatively was associated with worse PFS (hazard ratio [HR] 4.6, *p* = 0.00023).

Even in locally advanced disease, ctDNA may inform prognosis after curative-intent therapy. In a retrospective analysis of patients with stage III NSCLC treated with definitive chemo-radiation (CRT), Moding et al. found that patients with undetectable ctDNA after CRT had a good prognosis with or without consolidation immunotherapy [88]. By contrast, patients without a ctDNA response after CRT or rising ctDNA levels during durvalumab treatment had much poorer prognosis. This raises the question of whether those with ctDNA clearance post CRT could de-escalate therapy, or be spared consolidation immunotherapy, without compromising outcomes. Whereas those with rising or persistent ctDNA levels may be considered for intensification of therapy, or, perhaps even reassessment of surgical salvage (Fig. 1). These hypotheses will need to be tested and proven in prospective trials before they can be adopted as part of routine practice.

Recent studies have also demonstrated improved disease-free survival with adjuvant osimertinib in those with resected *EGFR* mutant lung cancer [109], and adjuvant atezolizumab in those with resected stage II-III PD-L1-positive NSCLC [114]. While these therapies are less toxic, they may still have serious side effects and significant costs. Understanding which patients are most likely to benefit from further therapy and which patients are already cured will continue to be an important question in the management of early-stage lung cancer.

Ongoing studies in early-stage lung cancer using plasma ctDNA for MRD detection are focused on identifying those at highest risk in order to accelerate drug development. One study, MERMAID-I (NCT04385368), compares outcomes with adjuvant chemo-immunotherapy with durvalumab versus chemotherapy alone in MRD-positive patients [115] Another compares outcomes on atezolizumab with or without a personalised vaccine (NCT04267237), and a third single-arm study explores the impact of adjuvant chemo-immunotherapy with atezolizumab on ctDNA clearance (NCT04367311). However, trials specifically addressing the value of ctDNA testing in treatment selection are needed in order to demonstrate the clinical utility of ctDNA testing post therapy and its impact on patient outcomes.

#### MRD in other solid tumours

In early-stage colorectal cancer (CRC), multiple studies have shown that the detection of MRD using plasma ctDNA is an important prognostic factor [116–120]. Investigators have used a range of fixed and personalised panels (e.g. Signatera bespoke mPCR NGS assay) to track tumour-specific SNVs and others. Tie et al. used the presence of post-operative ctDNA using an individualised panel to select patients for adjuvant therapy after resection of stage III colon cancer [121]. Based on previous research [117], they used an error reduction technology for detection of low-frequency mutations (Safe-Seq assay), to distinguish genuine mutations from artifactual variants. Patients underwent plasma ctDNA testing at 4-10 weeks post-surgery, if ctDNA was detected, they received adjuvant chemotherapy. If they experienced ctDNA clearance after 6 weeks of treatment, their estimated 3-year relapse-free interval was 77% versus 30% if ctDNA was still detectable post-adjuvant therapy (HR 6.8, *p* < 0.001). A larger randomised trial testing the impact of adjuvant chemotherapy on relapse-free survival in patients with ctDNA MRD after colon cancer resection has completed accrual and results are pending (NCT04058103), as are the results of other studies. A similar trial in patients with high-risk breast cancer with evidence of MRD post-resection has also completed accrual, exploring the benefit of adjuvant immunotherapy (NCT03145961).

In urothelial cancer, ctDNA analysis was used to assess response to adjuvant immunotherapy after surgical resection in patients from the IMvigor010 trial. After selecting 16 patient-specific clonal mutations using whole exome sequencing, a bespoke PCR assay was utilised to monitor ctDNA kinetics. Patients who were ctDNA positive at baseline (37% of patients), had an increased risk of recurrence (HR 6.3). In the atezolizumab arm, ctDNA positive patients had improved disease-free survival (HR 0.58) and OS compared to observation alone, and the immunotherapy arm also had higher rates of ctDNA clearance (18% vs 4%). There was no difference between arms if ctDNA was negative. This study supports the use of ctDNA to identify patients who would more likely benefit from adjuvant treatment, and in the future omitting the use of costly treatment in those who may be ctDNA negative [122].

ctDNA may provide a more sensitive and specific marker of disease activity. However, MRD detection still faces similar challenges to other tests in the screening setting. Because of the low concentration of plasma ctDNA, MRD assays require a high degree of analytical sensitivity that is often beyond current technical limits of mutation-based ctDNA detection methods [123].

### Cancer screening

The potential for ctDNA to improve current cancer screening processes is also under investigation, and the limitations of utilising the technology in these early disease settings applies to the use of ctDNA more broadly. Cescon et al. summarised the key attributes of a successful screening test in cancer [124]: high-sensitivity and specificity, low cost, ease of administration and minimal harm caused by the test. Liquid biopsy is easy (blood draw) and less invasive than other screening tests (e.g. colonoscopies). However, the capacity of liquid biopsy to reach the necessary sensitivity and specificity with acceptable cost remains the greatest challenge for its potential application in clinical practice [124].

First, sensitivity remains a major barrier, as ctDNA levels in early-stage disease are low due to limited tumour DNA shedding and limits on acceptable blood sample volumes. Tumour volume has been shown to correlate with plasma ctDNA variant allele frequencies (VAFs); therefore, if too small to be identified by current imaging methods, tumour mutations in plasma might be detected at VAFs below background sequencing error limits [123].

Second, a high specificity and positive predictive value are crucial in primary screening of a healthy population with low pre-test probability of cancer diagnosis. Detection of clonal hematopoiesis of indeterminate potential (CHIP)-related mutations can lead to false-positive results, limiting the clinical utility of detecting driver mutations in plasma cfDNA in this setting [14].

Third, most cancer mutations are not pathognomonic for a single tumour type, and therefore tissue-of-origin may be unclear. Finally, it is not clear what the clinical significance is following a positive result. ctDNA detected through liquid biopsy may never result in cancer or could be indolent, causing overdiagnosis of incidental cancers with direct and indirect harms [2]. Multiple groups are now developing potential assays that may enhance the positive predictive value of imaging alone [125]

#### Combination of cancer-specific mutations with other biomarkers

A combination of cancer-specific mutations with other biomarkers, such as circulating proteins, epigenetic alterations or viral sequences are also being explored as potential plasma-based screening biomarkers. Combined analyses of circulating proteins and cancer-associated mutations in plasma was used in the CancerSEEK platform [126]. Cohen et al. demonstrated the use of this platform for the diagnosis of eight common cancers in 1005 patients with non-metastatic, clinically detected tumours. Specificity was greater than 99% (only 7 of 812 young healthy controls scored positive), and sensitivity ranged from 69 to 98% (depending on cancer type). However, these results need to be validated in clinical trials with real-world controls, in which specificity and PPV may be much lower. Given that the study enrolled patients known to have underlying cancer, sensitivity in prospective studies is likely to be lower. Additionally, controls did not include individuals with all relevant co-morbidities, potentially overestimating specificity. Finally, an interventional study, as opposed to an observational study that does not report results to participants, is required to evaluate the impact of the test on patient management.

The DETECT-A study used a similar multi-cancer blood testing combined with PET-CT imaging to detect cancer in a prospective, interventional study of 10,006 women between 60–75 years not previously known to have cancer [127], in addition to standard of care screening test such as mammography. Positive plasma testing was independently confirmed by a diagnostic PET-CT, which also localised the tumour. Fifteen out of 26 patients with plasma positive disease underwent PET-CT imaging and 9 (60%) were surgically excised. Twenty-four additional cancers were detected by standard-of-care screening and 46 by neither approach. 1.0% of participants underwent PET-CT imaging based on false positive blood tests, and 0.22% underwent a futile invasive diagnostic procedure. These results showed the potential utility of multi-cancer blood testing combined with PET-CT for cancer screening; however, before integrating this screening tool into clinical practice, larger registration trials designed for regulatory approval are needed to confirm clinical utility, address risk vs. benefit to the screened population, and demonstrate cost-effectiveness.

Implementing ctDNA-based cancer screening into clinical practice will face both technical and biomedical challenges. Prospective trials need to demonstrate the clinical utility of these technologies in the screening setting, likely as a complementary tool in specific populations with high cancer risk (to address indeterminate results or increase clinical utility), and at an acceptable cost.

## Challenges and limitations

### Limitations

The limitations in sensitivity of detecting ctDNA depends on multiple factors, spanning the pre-analytical, analytical and post-analytical phases, as well as tumour biology. Factors that can impact the shedding of ctDNA and ultimately detection include the location of the primary tumour and metastatic lesions, disease burden, tumour stage, location, vascularity, cellular turnover, among others [128–130]. Sites of progression also influence plasma ctDNA levels, for example, patients with isolated intracranial disease are more likely to have undetectable plasma ctDNA [131]. These factors are amplified in the MRD and pre-diagnostic setting compared to the setting of established disease.

Several ctDNA assays used for molecular diagnosis use fixed panels targeting known cancer-associated genes. Others, developed for MRD detection, use a tumour-informed approach. While this approach may be more sensitive and even specific than use of a fixed panel, construction of individualised patient ctDNA panels requires more time and cost, and can only be performed in highly specialised laboratories. Fixed panels have faster turn around time, are less expensive and are more widely available. Thus, both types of assays remain of significant interest moving forward.

Single gene variant detection, broad NGS-based or multi-gene panels require laboratory-based quality optimisation. Each assay needs to demonstrate concordance with a gold standard, which can sometimes introduce confounding because of potential tumour heterogeneity. Some guidelines have encouraged the use of reference samples as they may offer greater reproducibility [12]. Recent cross-comparison studies have addressed the analytical performance of several assays at a range of VAFs [132, 133]. The Oncopanel Sequencing Working group tested five NGS-based ctDNA assays across several research and clinical centers using simulated experiments and reference ctDNA or mock cell-free DNA samples. VAFs at frequencies <0.5% showed less reliability and more variation in detection across the different platforms, although that threshold is evolving with newer technologies. DNA quantity was an important variable contributing to sensitivity, and the method of sequencing did not seem to influence overall performance of the assays. It is important that clinicians understand that at lower VAFs, variants may not be detected or may not be reproducible in patient samples. Optimal methods for analytical validation have been recently reviewed by Blood PAC and other international consortia [134]. Standardising these methods across laboratories and in guideline recommendations will be essential to ensure results are interpretable and consistent across laboratories.

The sensitivity of detection is improving with more novel NGS-based platforms, although these come at a higher cost and TAT. Limits of detection have improved with newer technologies such as CAPPSeq [135], iDES [136], and SAFE-seq, allowing detection of VAFs lower than 0.01% [137]. Currently available commercial and laboratory-developed assays are restricted to a set of selected genes (e.g. FoundationOne™, Guardant360™, MSK-ACCESS, among others), and use hybrid-capture based assays or Amplicon/PCR amplified-based assays. Most NGS-based platforms have demonstrated high specificity, whereas sensitivity appears to be a limiting factor with the proportion of false negatives approaching 30%. Hence guidelines routinely recommend that if plasma testing is negative, a tumour tissue biopsy should be pursued. More recently, shallow whole genome sequencing has emerged as a rapid, less costly and more easily accessible method of obtaining similar information from liquid biopsies [138]. This is under further exploration in multiple tumour types.

Detection of fusions has also presented some challenge, due to low prevalence of some gene fusions (e.g. *ROS1* prevalence at 1–2%) and larger sample requirements to increase DNA yields. Improved bioinformatics approaches and increased sequencing depth may overcome some of the limitations around fusion calling [139], as shown with improvements in the sensitivity and specificity of *ALK* fusion detection in the Non-invasive vs Invasive Lung Evaluation study [29]. Care must also be taken when interpreting ctDNA analysis which may not capture features such as histologic transformation, as seen in osimertinib resistance of *EGFR* mutant lung cancer with transformation to small-cell or squamous pathology [64, 140].

Molecular genotyping in advanced disease using plasma-based NGS panels has already been integrated into clinical practice in selected solid tumours, and clinical sensitivity has been demonstrated in prospective clinical trials in NSCLC [29] and also established by international groups such as the International Society of Liquid Biopsy (ISLB) [130]. In addition to ctDNA response, there are many other questions to be addressed, including optimal methods for bioinformatic analysis, whether absolute or relative changes in ctDNA levels are more informative and whether clearance is important, or reduction in levels alone is sufficient to inform treatment. The definition of a positive or negative test will also present challenges, if not standardised, with multiple studies utilising different methods [99]. The ability to identify relevant tumour variants versus leucocyte variants (clonal hematopoiesis) is also an important hurdle [141].

### Clinical utility

Despite encouraging data, the optimal way to integrate ctDNA strategies into clinical practice remains unclear. Prospective clinical trials are needed to explore whether changing therapy based on ctDNA dynamics prior to radiologic or clinical progression will improve outcomes compared to our current standard.

In resected colorectal cancer, ctDNA analysis using Signatera did not demonstrate benefit as a surveillance strategy over standard imaging. In addition, sensitivity of liquid biopsy was particularly poor for low volume lung-only disease recurrence [142]. Several prospective studies exploring the role of ctDNA for treatment modification in various tumour types are currently ongoing. For example, in the EORTC APPLE trial (NCT 02856893), patients with *EGFR* mutant lung cancer are randomised to receive either gefitinib followed by osimertinib based on progression detected by plasma ctDNA or detected by standard imaging [143]. Yu et al are recruiting patients to receive initial osimertinib; those with persistent ctDNA levels after 3 weeks will be randomised to continue osimertinib alone or addition of chemotherapy (NCT04410796). The CAcTUS study (NCT03808441) in patients with advanced *BRAF* mutant melanoma is a complex study planning to compare alternating targeted BRAF inhibition and immunotherapy based on ctDNA response (80% or more), compared to standard of care, using clinical or radiographic progression before switching treatments [144].

Future trials demonstrating the positive impact of ctDNA monitoring on patient outcomes are needed before widely integrating ctDNA into routine clinical care.

## Conclusion

The use of ctDNA adds value at diagnosis and at the time of clinical progression to identify resistant alterations in multiple tumour types. Future work is needed to demonstrate whether modifying therapy due to ctDNA changes before radiological progression improves survival outcomes in metastatic disease, and for MRD detection after curative-intent therapy. This may allow earlier or intensified interventions, potentially improving outcomes. Demonstrating the clinical utility of the various approaches will eventually lead to a sustainable increase in our ability to better prognosticate and cure patients with early-stage cancers. Prospective, well-designed clinical trials are now ongoing, and we eagerly await the data demonstrating improved clinical outcomes [3].

In the initial genotyping of advanced disease and detection of targeted therapy resistance in NSCLC cancer and other cancers, ctDNA is gradually being implemented into clinical practice. However, the largest impact of ctDNA will likely come from improving the management of earlier stage disease, including MRD detection, as well as improving detection of resistance in the metastatic setting. Trials that define the clinical utility of using plasma ctDNA are essential to moving oncology patient care forward.

## Data Availability

Data sharing is not applicable to this review article as no new data were created or analysed in this study. The authors confirm that the evidence supporting the conclusions of this study are available within the article and its references.
